# Psychometric properties of the Fraboni scale of ageism (FSA) applied to long-term caregivers in nursing homes

**DOI:** 10.1186/s12877-024-05229-1

**Published:** 2024-07-25

**Authors:** Jie Li, Ziyan Dong, Wen Xie, Liuqing Yang, Xiaojing Qi

**Affiliations:** 1 School of Nursing, Tongji Medical College, Huazhong University of Science and Technology, 13 Hangkong Rd., Qiaokou District, Wuhan, 430030 Hubei Province China; 2 Department of Nursing, Peking Union Medical College Hospital, No.1 Shuaifuyuan Wangfujing Dongcheng District, Beijing, 100730 China

**Keywords:** Fraboni scale of ageism, Long-term care, Reliability, Validity, Psychometric properties

## Abstract

**Background:**

The aging society has resulted in enormous demand for long-term care services. However, ageism is a common phenomenon in long-term care facilities, which not only hinders the quality of care for the recipients but also negatively influences caregivers’ well-being. In this paper, we first applied the Fraboni Scale of Ageism (FSA) to evaluate its reliability and construct validity among Chinese long-term caregivers in nursing homes. This study could contribute to assessing the prevalence of ageism in Chinese long-term caregivers, prompting facilities and the government to recognize the issue of ageism and explore necessary interventions to reduce ageism in long-term caregivers.

**Methods:**

This is a cross-sectional study. We recruited 392 long-term caregivers using a convenience sampling strategy in nursing homes from two cities in Chinese central and northern regions. Parameters included the demographic characteristics, Cronbach’s alpha coefficients, and intraclass correlation coefficient. The construct validity was conducted by exploratory factor analysis and confirmatory factor analysis.

**Results:**

The Cronbach’s alpha of FSA (Chinese version) was 0.856 and ICC was 0.871. The factor analysis identified 3 principal factors, explaining 43.95% of the total variance. The 3-factor model was confirmed to fit by confirmatory factor analysis.

**Conclusions:**

The findings confirm that the FSA is easy to use and has good psychometric properties. This study will contribute to improving the condition of ageism, thereby improving the quality of care for the elderly and retention of professional talents in the LTC system.

**Supplementary Information:**

The online version contains supplementary material available at 10.1186/s12877-024-05229-1.

## Background

Population aging has been a global phenomenon, especially in China. China is classified as one of the most rapidly aging countries and has the largest aging population in the world [1]. It’s reported that elderly people with disabilities have accounted for 24% of the whole elderly population in China because of the longer human life expectancy [2]. Long-term care (LTC) for disabled elders is becoming an important challenge for families and the whole nation.

LTC refers to a set of activities provided by informal caregivers (i.e., family, friends, and volunteers) and professional staff to help people with significantly reduced intrinsic capacity and functions in settings like homes, community centers, and LTC facilities [3]. Elderly individuals residing in long-term care facilities often exhibit frailty, multiple co-morbidities, and sometimes disabilities, and their daily care and well-being primarily rest in the hands of professional caregivers, so long-term caregivers in nursing homes are usually vocational nursing employees hired by the LTC facilities [4, 5]. China aims to establish a multi-level elderly service system based on home, supported by communities, and supplemented by LTC facilities according to the “13th Five-Year Plan for Economic and Social Development of the People’s Republic of China” [6]. However, as women’s employment rate increases, demographic and socioeconomic characteristics shift, and the relationship between older and younger generations becomes more estranged from each other [7], LTC for older adults with disabilities is becoming a challenge for families. It seems increasingly impractical to depend solely on families to take care of older adults. On the other hand, home and community-based services remain spotty [8]. As a result, these phenomena make public formal care facilities, such as nursing homes (NHs), become an indispensable source of social support. As the main members of NHs, long-term caregivers who represent the core workforce in dealing with the aging problem of China, are also facing a significantly increased need [9].

Ageism is a term to describe the systematic stereotype and discrimination against older people because of their older age [10]. It is a set of beliefs, attitudes, norms, and values used to justify age-based discrimination [11]. It can be manifested in both positive and negative stereotypes, as well as in prejudice against (or to the benefit of) the elderly [12]. For example, it might be reflected in using words that deny and humiliate the elderly [13], or supposing that all older people are wiser and happier than young people [11].

We must acknowledge that people living in NHs do need strong adaptability because of the high level of renunciation of their former life. It will further reduce the quality of life of the elderly if they also suffer ageism again. Ageist beliefs often cause exclusion in decision-making and inhibit opportunities to practice autonomy for the elders [14]. This stereotype is also present in the Chinese healthcare system [15], resulting in the elderly being often disadvantaged and unfairly treated [16].

Ageism is a common phenomenon that is found in many domains. In LTC facilities that provide care for the elderly, this phenomenon deserves even more of our attention. It can not only hinder the quality of care for the recipients but also negatively influence the caregivers’ well-being. It is essential to focus on ageism in LTC facilities because of the following reasons. Firstly, an increasing need for LTC service is expected in the future due to population aging and the expansion of disability trends [2]. Secondly, the people living in LTC facilities tend to be vulnerable, as the majority of them have trouble carrying out activities of daily living [17]. They are at high risk of becoming victims of ageist attitudes and behavior [18]. Finally, there is evidence indicating that long-term caregivers frequently have low qualifications [19]and low wages [20] which are connected with higher levels of ageist attitudes and stereotypes [21, 22]. Ageist behaviors and stereotypes may contribute to long-term caregivers’ reluctance to stay with old people [23]. Therefore, ageism may affect their willingness to take care of the elderly [15]. 

There are several tools frequently used to measure the prevalence of ageism. Kogan’s Attitudes Toward Old People Scale (KAOP) includes 34 items applying a 6-point Likert scaling, with 17 negative and 17 positive responses. It had confirmed that KAOP had good reliability and validity [11]. Relating Older People Evaluation (ROPE), an indirect measure of negative and positive ageism contains 20 items on a 3-point scale, mainly used to assess individuals’ behaviors that people may engage in during daily life [15, 24]. However, this scale cannot measure the extent of ageism attitude or emotion. The Fraboni Scale of Ageism (FSA) was developed based on the concept of ageism proposed by Butler [25] and has 29 items assessed on a 4-point scale. The instrument is commonly applied to estimate the cognitive status of ageism. Of the instruments above, KAOP mainly pays attention to the measurement of stereotypes of older adults; ROPE only estimates the individual ageist behavior for older adults; FSA evaluates both cognitive and affective aspects of ageism. And it consists of 3 multidimensional constructs: antilocution, avoidance, and discrimination [25, 26]. It is beneficial to assess the cognition and emotions of long-term caregivers towards the elderly in LTC sectors, which can measure the level of ageism more comprehensively and intuitively to reduce the prevalence of ageism in LTC facilities further.

The scale of FSA has been used in many fields at home and abroad, such as in healthy people and healthcare workers in hospitals [14, 27]. We have previously cross-culturally adapted the FSA into a Chinese version and applied it to the medical student population, showing good reliability and validity [28]. However, it must be admitted that medical students and long-term caregivers are completely different groups. Firstly, long-term caregivers in Chinese LTC facilities tend to be middle-aged and older women who have been laid off, retired, or migrated to cities to work [29]. They are older and less educated than medical students. Besides, long-term caregivers have more direct and constant contact with older adults in comparison with students. Each of these characteristics may influence the expression of ageism among long-term caregivers.

Therefore, the purpose of the study was to (1) determine FSA’s reliability and validity among caregivers in LTC facilities in Chinese cultural contexts and (2) to explore group differences in ageism, specifically, we tested the association between age and years of work experience and ageism as well as differences in ageism scores by education level, marital status, gender, and managerial roles. To our knowledge, this is the first time that the 29-item Chinese version of FSA has been tested on a sample of long-term caregivers.

## Methods

### Participants

Long-term caregivers in NHs facilities were recruited to participate in this cross-sectional study. The study was carried out from June 2021 to June 2022 in Wuhan, Hubei province, and Kaifeng, Henan province. The inclusion criteria were as follows: (1) caregivers who directly take care of the elderly, (2) have been engaged in the elderly service for>3 months, and (3) have volunteered to participate in this survey. The exclusion criteria were: (1) caregivers who are unable to complete the questionnaire due to study or vacation, and (2) are not willing to participate in the study.

### Data collection

Approval was obtained from the Ethics Committee of the Tongji Medical College of Huazhong University of Science and Technology (approval number S028) and written informed consent was obtained from the subjects. The study participants were recruited based on the above inclusion and exclusion criteria using a convenience sampling strategy from 57 NHs in Wuhan, Hubei Province, and Kaifeng, Henan Province, China. The way of on-site question and answer was employed by trained investigators to collect data because of the universal older age and lower education level with long-term caregivers. Additionally, since test-retest reliability usually is conducted during an initial pilot study involving only a small sample [30], we selected 25 subjects to fill in the same questionnaires 2 weeks following the first time.

### Instrument

#### Demographic characteristics

Demographic characteristics included age, gender, marriage, position, education level, method of appointment, and years of work.

#### The Fraboni scale of ageism

The Chinese-translated version of the FSA was supplied by Junyao Fan [28], who is an important member of our research group and represents the copyright owners of the FSA (Chinese version). We used the Chinese version of the FSA, which consists of 29 items assessing the strength of an individual’s ageism to older people. The original version of FSA contains three subscales: antilocution, avoidance, and discrimination [25]. Item numbers 2, 8, 12,14, 21, 22, 23, 24 are positive statements and scores should be reversed. Scores for each item range from 1 (strongly disagree) to 4 (strongly agree) on a Likert-type scale. After reversing these positive items, the total scores ranged from 29 to 116, with higher scores indicating greater ageism. The questionnaire took approximately 6 min to complete.

### Data analysis

Item analysis was computed by the critical ratio and based on the psychometric calculation of item differentiation, we chose 27% as the ratio for the high and low groupings [31]. Participants were divided into upper and lower groups comprised of the top 27% and bottom 27% according to the highest and lowest scores of FSA. Items will show good discrimination if significant differences exist between each item in the upper and lower groups in the independent sample t-test.

Internal consistency reliability was evaluated using Cronbach’s coefficient and item-to-total score correlations. The acceptable value is set at>0.70 [32]. The scale was surveyed twice with an interval of about 2 weeks among 25 subjects to assess the test-retest reliability, which reflects the time durability by computing the intraclass correlation coefficient (ICC). The values<0.5, 0.5–0.75, 0.75–0.9, and>0.9 indicate poor reliability, moderate reliability, good reliability, and excellent reliability respectively [33].

The exploratory factor analysis (EFA) and confirmatory factor analysis (CFA) were used to evaluate construct validity. The sample size for CFA is ≥ 200, we chose 210 samples to test CFA and the remaining 182 samples to test EFA [34]. The samples are suitable for factor analysis if the Kaiser-Meyer-Olkin (KMO) is > 0.60 and Bartlett’s test of sphericity is significant. Factors with eigenvalues>1 were selected, while items with the maximum factor loading value >0.45. CFA was used to test the model fitness. The chi-square degree of freedom ratio (CMIN/DF) < 3, the compare fitting indices (CFI) > 0.90, the incremental fit index (IFI) > 0.90, the Tucker-Lewis index (TLI) > 0.90, the normative fitting indices (NFI) > 0.90, and the root-mean-square error of approximation (RMSEA) < 0.1 indicate the model fit well [34].

Mean values and standard deviation (SD) (for symmetric distribution) or median and quartiles (for skewed distribution) were calculated for continuous variables, while frequency and percentage were used for categorical variables. Spearman’s correlation coefficients, the Wilcoxon rank-sum test, and the Kruskal-Wallis H test were calculated to examine the association between demographic features and ageism scores.

Statistical analysis was performed by the IBM SPSS software, version 21.0. *P* < 0.05 was considered to be statistically significant. CFA was carried out using SPSS Amos, version 21.0.

## Results

### Demographic characteristics

While 396 long-term caregivers in Chinese NHs participated in this study, 4 didn’t finish the survey because of temporary service for the elders or other critical affairs. As a result, a total of 392 subjects completed the questionnaire. The sample consisted of 350(89.3%) female, 42(10.7%) male. Of which 30.1% had a management position, half of them were just temporary workers. The median(M) and interquartile range (IQR) of age and working years were 52(46, 56), and 5(3, 7). In terms of educational attainment, merely 11.7% of them graduated from junior college, and 5.4% of them had acquired undergraduate degrees. Junior high school (40.1%) and married status (87.5%) were the most prominent composition in NHs. In addition, our sample was skewed, with participants’ FSA scores averaging 2.10.

### Item analysis

We found item 16 (“Most elderly people should not be allowed to renew their drivers licenses”) was the only one that had no statistical significance (t=-1.213, *p* = 0.226) in the independent sample t-test, which indicates that discrimination between the upper and lower groups on this item was poor. It was suggested to be eliminated. The results of the item analysis are presented in Additional File 1.

### Validity

#### Construct validity

**Fig. 1:**
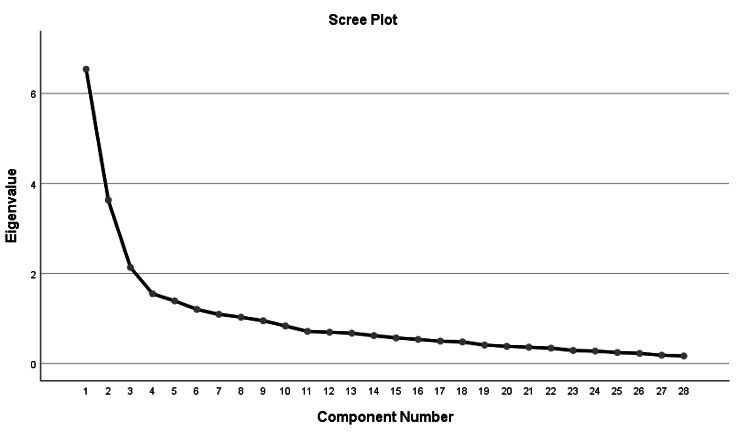
Scree slope plots of eigenvalues for FSA

With principal component extraction and maximum variance method in the EFA (for 28items), The KMO = 0.786 and Bartlett’s test of sphericity was significant (*χ2* = 2106.881, *p* < 0.001), supporting factor ability of the correlation matrix. The first EFA yielded an 8-factor structure with eigenvalues above 1, explaining a variance of 66.31%. However, inspection of the scree plot suggested that the curve flattened after the third factor (see Fig. 1), thus we chose three factors that are consistent with the original FSA. Then the second-factor analysis was performed and the factor number was limited to 3, which divided into avoidance, excluded, and stereotypes explaining 43.95% of the variance. The first factor was composed of 12 items referred to rejection to accept older people in activities, which was identified as “excluded”. The second factor was defined by 10 items that showed a bad impression of old people, thus representing “stereotype”. The third factor contained 6 items entirely related to reluctance to interact with older people, so this factor was generally labeled as “avoidance”. Table 1 shows the results of the exploratory factor analysis and the factor structure of the other versions for explicit comparison. Item 22 was recommended to be removed because the load on each factor was < 0.45.

**Fig. 2:**
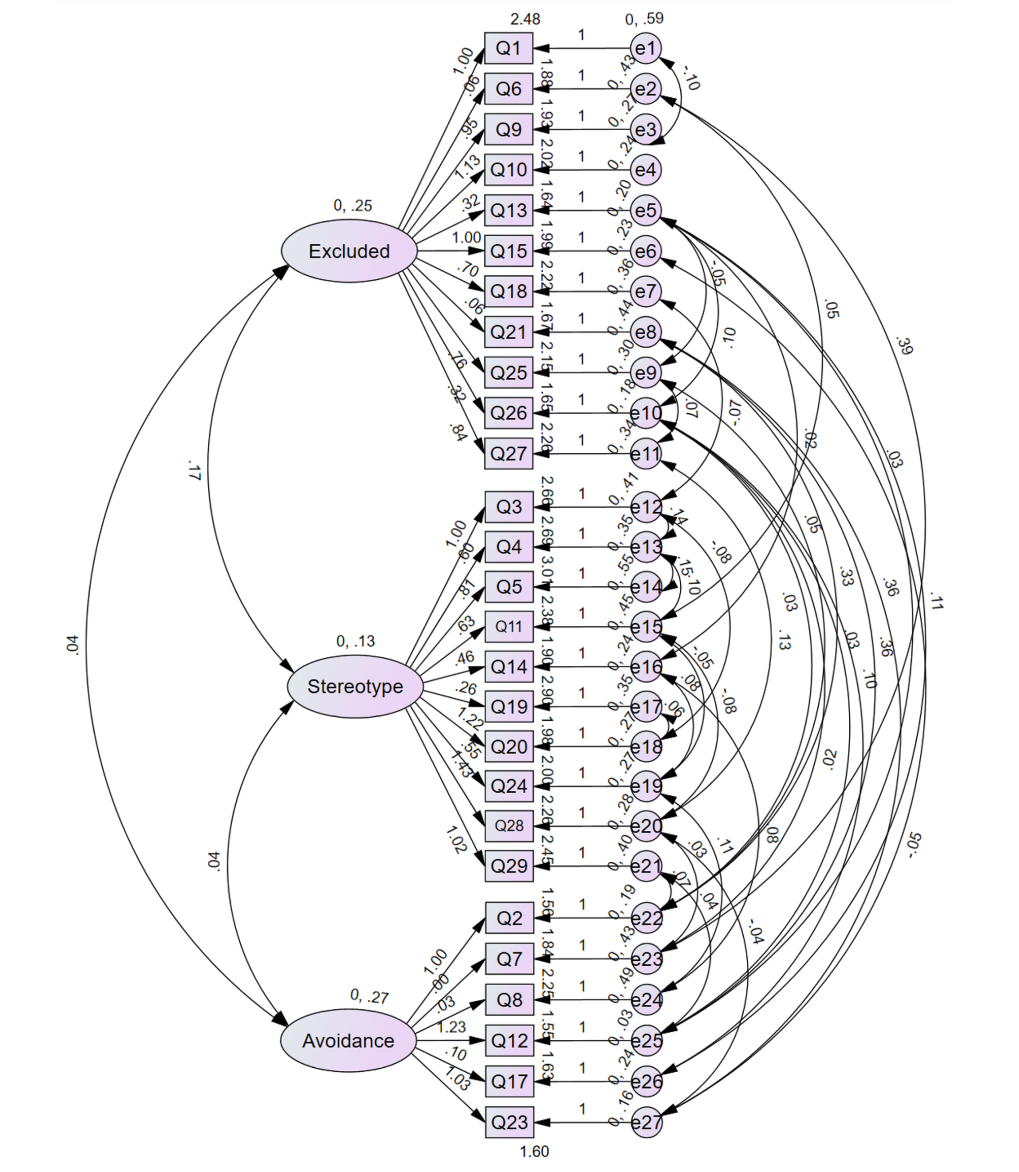
A confirmatory factor model of the Chinese version of Fraboni Scale of Ageism

Then CFA was performed to evaluate the three-factor model (see Fig. 2). The model was modified according to the Modification Indices. In the revised model, the CMIN/DF was 1.745; CFI was 0.919; IFI was 0.921; TLI was 0.901, and RMSEA was 0.060. Generally, the results of EFA and CFA indicated that the structural validity of FSA (Chinese version) was acceptable.

**Table 1:** Item loading for principal component factor analysis and original scale dimension (*n* = 392)

Item no.	Factor and loading			Original scale dimension	Rupp et al. (USA)	Bodner & Lazar (Israel)	Kutlu et al. (Turkey)
	Excluded	Stereotype	Avoidance				
**10.** Feeling depressed when around elderly people is probably a common feeling	**0.797**	0.235	0.093	Avoidance	Separation	Avoidance	Discrimination
**15.** I personally would not want to spend much time with an elderly person	**0.740**	− 0.009	0.059	Avoidance	Affective att.	Avoidance	Avoidance
**9.** Complex and interesting conversation cannot be expected from most elderly people	**0.695**	− 0.093	0.244	Antilocution	Separation		Discrimination
**18.** Most elderly people should not be trusted to take care of infants	**0.663**	− 0.097	0.070	Discrimination	Stereotypes	Avoidance	Stereotypes
**26.** I would prefer not to live with an elderly person	**0.645**	− 0.040	0.164	Avoidance			Avoidance
**13.** I would prefer not to go to an open house at a seniors club if invited	**0.597**	− 0.064	0.147	Avoidance	Stereotypes	Contribution	Avoidance
**6.** I sometimes avoid eye contact with elderly people when I see them	**0.585**	− 0.108	0.229	Avoidance	Separation		Avoidance
**21.** The company of most elderly people is quite enjoyable	**0.537**	− 0.129	0.274	Discrimination	Affective att.	Contribution	Avoidance
**1.** Teenage suicide is more tragic than suicide among the elderly	**0.499**	0.068	0.076	Antilocution	Stereotypes	Stereotype	Stereotypes
**27.** Most elderly people can be irritating because they tell the same stories over and over again	**0.489**	0.418	− 0.101	Antilocution		Contribution	Stereotypes
**25.** Most elderly people would be considered to have poor personal hygiene	**0.451**	0.377	0.093	Antilocution		Avoidance	Stereotypes
**22.** It is sad to hear about the plight of the elderly in our society these days	0.431	0.027	0.376	Discrimination	Affective att.	Contribution	
**11.** Elderly people should find friends their own age	− 0.203	**0.727**	− 0.046	Avoidance	Separation	Avoidance	Discrimination
**3.** Many elderly people are stingy and hoard their money and possessions	0.047	**0.602**	− 0.183	Antilocution	Stereotypes	Stereotype	Stereotypes
**4.** Many elderly people are not interested in making new friends, preferring instead the circle of friends they have had for years	0.042	**0.580**	− 0.139	Antilocution	Stereotypes	Contribution	Stereotypes
**19.** Many elderly people are happiest when they are with people their own age	− 0.307	**0.567**	0.019	Avoidance	Stereotypes	Contribution	Stereotypes
**29.** Elderly people do not need much money to meet their needs	− 0.035	**0.560**	0.057	Antilocution		Avoidance	Stereotypes
**5.** Many elderly people just live in the past	0.041	**0.552**	0.017	Antilocution	Stereotypes	Stereotype	Stereotypes
**28.** Elderly people complain more than other people	0.411	**0.483**	0.101	Antilocution			Stereotypes
**24.** Most elderly people are interesting, individualistic people	− 0.163	**0.481**	0.464	Discrimination			Avoidance
**20.** It best that elderly people live where they won’t bother anyone	0.457	**0.480**	0.328	Discrimination	Affective att.		Discrimination
**14.** Elderly people can be very creative	0.056	**0.471**	0.325	Avoidance	Separation	Avoidance	Avoidance
**17.** Elderly people don’t really need to use our community sports facilities	− 0.009	− 0.007	**0.726**	Discrimination	Separation		Avoidance
**23.** Elderly people should be encouraged to speak out politically	0.280	− 0.036	**0.715**	Discrimination	Affective att.	Avoidance	Discrimination
**12.** Elderly people should feel welcome at social gatherings of young people	0.254	− 0.282	**0.653**	Avoidance	Separation	Avoidance	
**8.** Elderly people deserve the same rights and freedoms as other members of our society	0.222	0.104	**0.601**	Discrimination	Stereotypes	Stereotype	
**7.** I don’t like it when elderly people try to make conversation with me	0.417	− 0.197	**0.545**	Avoidance	Separation	Stereotype	Avoidance
**2.** There should be special clubs set aside within sports facilities so that the elderly can compete at their own level	0.184	0.142	**0.519**	Discrimination	Stereotypes	Stereotype	

Extraction method: principal component analysis

Rotation method: Kaister standardized orthogonal rotation

a rotation converges after five iterations

#### Reliability

Item 22 was excluded after the factor analysis and item 16 was removed after the item analysis. Therefore, the final Chinese version of FSA included 27 items.

The Cronbach’s alpha for the total score of FSA was 0.856, and the subscales of avoidance, excluded, and stereotype varied from 0.835 to 0.709. The ICC value was 0.871, and the subscales ranged from 0.536 to 0.907, indicating that internal consistency reliability and retest reliability of the Chinese version of FSA were acceptable.

#### Relationship of participants’ characteristics with FSA scores

As shown in Table 2, statistically significant negative correlations between the FSA scores with age (ρ=-0.146, *p*<0.01) and years of work (ρ=-0.282, *p*<0.01) were found. Table 3. shows the results of the analysis of differences in FSA scores by gender, marital status, level of education, and managerial roles assumed. FSA scores differed significantly by marital status (*p*<0.01) and managerial roles (*p*<0.05), but not by gender or education level.

**Table 2:** Correlation between FSA scores with age and years of work (*n* = 392)

	Rho	*p*
Age	− 0.146	**0.004**
Years of work	− 0.282	**0.000**

**Table 3:** Comparison of FSA scores with gender, marital status, education, and managerial roles (*n* = 392)

	*N* (%)	z^a^ H^b^	*p*
**Gender**		.464^a^	0.643
Female	350(89.3)		
Male	42(10.7)		
**Marital status**		12.712^b^	**0.005**
Single	14(3.6)		
Married	343(87.5)		
Divorced	3(0.8)		
Widowed	32(8.2)		
**Education**		4.760^b^	0.313
Primary and below	78(19.9)		
Middle school	157(40.1)		
Senior high school	90(23.0)		
Junior college	46(11.7)		
Bachelor’s degree or above	21(5.4)		
**Managerial Roles**		2.297^a^	**0.022**
Yes	118(30.1)		
No	274(69.9)		

Note: p = significance level

^**a**^ **z (Wilcoxon rank-sum test)**

^**b**^ **H (Kruskal-Wallis H test)**

## Discussion

In general, the findings of the reliability, validity, and usefulness proved that the FSA was satisfactory as a practical tool for evaluating ageism among long-term caregivers in Chinese NHs. The method conducted in the study followed the approach of validating the same psychometric instruments in other studies [28]. The scale is of great importance considering that the available tools specifically designed to test ageism among long-term caregivers are still limited currently.

There were eight factors whose eigenvalues>1 in the factor analysis, but we thought it was difficult to make them meaningful and name them. Therefore, according to the characteristics of the scree plot, the second EFA identified 3 specific structures that reflected the heterogeneity and wide spectrum of discrimination for the elderly, which resembled the original scale. However, the items contained in the factor structure somewhat differed from the original scale by Fraboni [25]. Additionally, there were also a few disparities in the factor structures among the versions of the USA, Turkey, Israel, and China. The adapted versions coincidentally all discard the label of antilocution, and the avoidance factors are broadly similar. The U.S. version of the FSA takes the conceptual connotations and divides the factor structure in a way that emphasizes the affective (separation and affective attitudes) and cognitive (stereotype rather than antilocution) components of the measurement of ageism [26]. The Turkish version adopted the psychological property of stereotype and remained consistent with the original scale in the avoidance and discrimination components [35]. The Israel version differs by interpreting the discrimination component of the FSA as a negative perception of older people’s contribution to society and therefore naming contribution [27], while the Chinese version visualizes discrimination as the denial of older people’s participation in activities and names it as excluded [28]. Admittedly, stereotype is a more understandable term, and the different expressions of the other factors are adapted to the linguistic expressions of different countries. The difference in items included in each factor may be related to population and cultural background variations.

There was no statistical significance in the item analysis for item 16 “Most old people should not be allowed to renew their driver’s licenses”. Although the upper age limit for driver’s licenses was adjusted from 60 to 70 years old in China, there are still some concerns about the safety of elderly drivers due to their declining eyesight and cognitive capability. Thus, the inconsistent attitudes on whether the elderly should be allowed to obtain driver’s licenses may explain the elimination of this item. The only item that didn’t reach the lowest threshold of factor load was the 22nd item, which was related to the attitudes towards the situation of the elderly belonging to the structure of “discrimination” in the original tool. When it comes to the situation of the elderly, the subjects were not very clear about what this referred to and needed exact explanations from the researchers. However, part of the questionnaire for this study was collected online and was not explained face-to-face. The item was tendentious in the original scale, i.e., it specifically referred to the plight of the elderly. Therefore, the unawareness of the situation of the elderly together with the ambiguities of this problem may explain the low factor load of the item. This should be improved in the Chinese context in our future research so that the tendency of the item can be more clearly stated.

Besides, results from the present study revealed that the item numbers and factor structure were somewhat different from the previous validations among medical students. The current finding retained 27 items (items 16 and 22 were deleted), while previous validations retained 22 items and deleted six items (1, 2, 8,12, 22, and 29) because of the low total correlation of items [28]. As we mentioned earlier, caregivers are very distinct from students in terms of age, education level, work experience, and contact experience with the elderly. The fact that there are disparities in psychometric properties might be related to the population differences between long-term caregivers and students. Consequently, further research among various populations on the FSA is suggested.

The average score of ageism among long-term caregivers was at a lower middle level. This demonstrated that the attitudes of long-term caregivers toward the elderly are generally acceptable in China. On the one hand, this may be explained by the fact that the majority of the participants are female. Women tend to socialize into caregiving roles in the family, making them more empathetic to seniors. On the other hand, this may be related to the collectivist culture practiced in East Asia. Eastern cultures, such as China, Israel, and Turkey, are collectivism-oriented, emphasizing respect and filial piety for older persons. In particular, China has always respected the culture of Confucianism and emphasized that filial piety is the first of all good deeds [36]. Especially for relatively older long-term caregivers, this perception is deeply ingrained. Western society, on the other hand, is individualistic, with a popular culture of chasing youth, freedom, and active living, as well as the ideal of successful aging, and there is a fear of failure, death, and aging. This fear manifests itself in stigmatization and aversion to older people who do not meet the criteria for success, avoidance of older people, and ageism [37]. It is this discrepancy that derives the assertion that age discrimination is less prevalent in Eastern societies. The root behind culture is the economy, and with the economic takeoff comes consumerism, which by its very nature is anti-aging [38]. With China’s economic growth and urbanization in recent years, young people don’t necessarily have a more positive attitude towards old age than those from Western cultures. Considering that even though they share the same oriental culture, there are huge differences between East Asian countries. The application of our results to other environments and other populations also needs to be validated.

When the FSA scores were considered by demographic features, only the marriage, years of work, Managerial roles assumed, and age explained the between-group differences. The years of work have been found to correlate with the total score in the study which was in line with the previous studies [39, 40]. Years of work were negatively correlated with total scores on the FSA. The reason may be that these long-term caregivers have been in contact with the elderly for a long time, so they will have a better understanding of the disease and personality characteristics of the elderly and tend to have a more positive attitude towards them.

Age was negatively related to ageism against the elderly. The older the long-term caregiver is, the lower the total score is. This is most likely because older caregivers are similar to the recipients in age. They tend to understand the feelings of older adults and are more likely to empathize with them. Additionally, caregivers with management positions had less ageism than those without management positions. Some of the managers in private NHs that were less profitable participated and often stated that they persisted because of compassion and love. On the other hand, most caregivers in managerial positions generally have a strong sense of responsibility [41]. Therefore, caregivers who have management positions are less likely to discriminate against older adults.

Married, divorced, and widowed caregivers all had lower scores than unmarried individuals. Caregivers who had marital experience were less likely to discriminate against older adults possibly because married women are likely living in large families where they are responsible for taking care of the elderly. Previous studies have shown that people who live in large families often have less age discrimination [42]. In addition, widowed caregivers have the least ageism compared to all marital statuses. Most of them are widowed in middle or old age, so they are more friendly to the elderly and even put their emotions to work.

However, this study didn’t find any difference in the level of ageism by gender and education, which is not consistent with the findings of the literature [11, 42]. Subjects in NHs facilities cared for the old population, which was different from other contexts. Thus, the influence of individual characteristics on ageism was not significant. Additionally, it may be related to the large difference of gender in the sample size (89.3% of women) and the small sample size of high education (only 5.4% of college and above).

Other findings related to the FSA indicated that this instrument was well accepted by most of the subjects because it was easy to conduct and needed less mean amount of time to finish [26–28, 35, 42, 43]. They were also been further substantiated in this research. Methodologically, it’s a critical element to consider the acceptability of a tool in the target population, which is particularly important for the practical application of evaluation tools in clinical practice.

It is obvious that ageism was one of the main factors that prevented caregivers from providing care to the elderly in LTC facilities. Less negative ageist behaviors will increase their willingness to care for older adults [15]. Moreover, caregivers who have positive ageist behaviors are usually more likely to stay [44]. Focusing on the ageism of LTC workers may be of particular significance in the task of talent maintenance of caregivers and quality of care for old adults. Previous studies indicated that interventions that combine elements of education and intergenerational contact have a positive impact on attitudes towards older people, while most evidence comes from Western countries [45]. Aging education and intergenerational contact interventions are usually conducted in student populations by providing knowledge and information about aging as well as extended contact to reduce stereotypes and weaken ageism [46]. There is also a need for aging education and training measures for the long-term caregivers, especially for younger employees, which requires efforts by health-care institutions and administrators of LTC facilities. In addition, future research needs to validate the effectiveness of the above mainstream interventions in collectivist Eastern countries and explore more effective intervention programs to address the crisis of ageism.

### Limitations

There are several limitations in the current study. Firstly, due to human and financial constraints, we employed a convenience sampling strategy in only two cities, which restricts the generalizability of our findings. Specifically, our sample was drawn exclusively from central and northern China, regions characterized by moderate levels of economic development, which may lead to selection bias. Besides, nearly four hundred participants were recruited, with female employees constituting the majority, while participants with bachelor’s degrees or higher were underrepresented. Although existing data indicated that caregivers in long-term care settings are predominantly female and possess lower overall educational attainment [47], the specific proportions within our sample may vary. This variability impedes a comprehensive exploration of ageism from diverse perspectives and may impact the generalizability to other countries and regions. Additionally, we conducted only a preliminary analysis of the relationship between ageism and demographic characteristics among Chinese long-term caregivers in nursing homes. Unfortunately, we did not comprehensively capture other critical underlying factors related to employees’ work experiences.

To address these limitations, future research should not only expand sample sizes across multiple regions but also employ sampling methods that enhance sample representativeness. Furthermore, considering employee work experiences (such as job satisfaction and burnout) will be essential for a more comprehensive exploration of ageism within the elderly care worker population.

## Conclusion

In conclusion, the Chinese version of the FSA has been found to be comparable to the original version in psychometric properties. Its reliability and validity had also been verified among long-term caregivers, which supported FSA and could be applied for measuring the ageism of caregivers in Chinese LTC facilities. This study will contribute to improving the status of ageism, thereby improving the quality of care for the elderly and retention of professionals in the LTC system.

### Electronic supplementary material

Below is the link to the electronic supplementary material.

Supplementary Material 1

## Data Availability

The datasets analyzed during the current study are available from the corresponding author on reasonable request.

## Abbreviations

FSA: Fraboni Scale of Ageism
LTC: Long-term Care
NHs: Nursing Homes
KAOP: Kogan’s Attitudes Toward Old People Scale
ROPE: Relating Older People Evaluation
ICC: Intraclass Correlation Coefficient
EFA: Exploratory Factor Analysis
CFA: Confirmatory Factor Analysis
KMO: Kaiser-Meyer-Olkin
CMIN/DF: The chi-square degree of freedom ratio
CFI: Compare Fitting Indices
IFI: Incremental Fit Index
TLI: Tucker-Lewis Index
NFI: Normative Fitting Indices
RMSEA: Root-Mean-Square Error of Approximation
M: Median
IQR: Interquartile Range
