# Supplementary material for: Psychometric properties of the Fraboni scale of ageism (FSA) applied to long-term caregivers in nursing homes
Source: BMC Geriatr. 2024 Jul 25;24:632. doi: 10.1186/s12877-024-05229-1 (PMC11270974; doi:10.1186/s12877-024-05229-1)
Supplement: Supplementary file 1 — Supplementary Material 1 [file 12877_2024_5229_MOESM1_ESM.docx]

| Item No. | t | p | Item No. | t | p |
| --- | --- | --- | --- | --- | --- |
| 1 | -11.080 | .000 | 16 | -1.213 | .226 |
| 2 | -7.248 | .000 | 17 | -7.593 | .000 |
| 3 | -6.671 | .000 | 18 | -9.538 | .000 |
| 4 | -6.430 | .000 | 19 | -2.843 | .005 |
| 5 | -6.923 | .000 | 20 | -15.365 | .000 |
| 6 | -11.498 | .000 | 21 | -5.282 | .000 |
| 7 | -11.056 | .000 | 22 | -6.311 | .000 |
| 8 | -5.706 | .000 | 23 | -6.161 | .000 |
| 9 | -12.813 | .000 | 24 | -5.963 | .000 |
| 10 | -16.857 | .000 | 25 | -11.026 | .000 |
| 11 | -5.290 | .000 | 26 | -11.200 | .000 |
| 12 | -7.066 | .000 | 27 | -10.238 | .000 |
| 13 | -11.560 | .000 | 28 | -13.370 | .000 |
| 14 | -7.441 | .000 | 29 | -6.941 | .000 |
| 15 | -11.734 | .000 |  |  |  |

**Additional file 1: Results of item analysis**
